# Efficacy and safety of outpatient fludarabine, cyclophosphamide, and rituximab based allogeneic hematopoietic cell transplantation in adults with severe aplastic anemia

**DOI:** 10.1038/s41409-024-02323-1

**Published:** 2024-06-15

**Authors:** Rachel M. Gilmore, Karin Abernathy, Kendall Shultes, Dwight D. Eplin, Lindsay Orton, Adetola Kassim, Salyka Sengsayadeth, Wichai Chinratanalab, Tae Kon Kim, Bhagirathbhai Dholaria, Reena V. Jayani, Bipin N. Savani, Elizabeth McNeer, Leena Choi, Katie Gatwood

**Affiliations:** 1 Cleveland Clinic, Department of Pharmacy, Cleveland, OH USA; 2 Sarah Cannon Cancer Center, TriStar Centennial Medical Center, Nashville, TN USA; 3 VA Tennessee Valley Healthcare System, Nashville, TN USA; 4 US Dept of Veterans Affairs, VISN 10 Clinical Resource Hub, Nashville, TN USA; 5 Vanderbilt University Medical Center, Department of Pharmaceutical Services, Nashville, TN USA; 6 Vanderbilt University Medical Center, Division of Hematology/Oncology, Nashville, TN USA; 7 Tennessee Valley Healthcare System Stem Cell Transplant and Cellular Therapy Program, Nashville, TN USA; 8 Vanderbilt University Medical Center, Department of Biostatistics, Nashville, TN USA

**Keywords:** Anaemia, Chemotherapy

## Abstract

The age effect in severe aplastic anemia (SAA) following allogeneic hematopoietic cell transplantation (HCT) favors the use of reduced intensity conditioning (RIC) regimens in older adults. We implemented a non-myeloablative regimen consisting of fludarabine, cyclophosphamide, and rituximab (FCR) to improve HCT outcomes in SAA. Patients who underwent first HCT for SAA utilizing an FCR regimen between January 2016 and May 2022 were included. Outcomes analyzed included time to engraftment, incidence of graft failure, GVHD, viral reactivation, disease recurrence, and GVHD-free, relapse-free survival (GRFS). Among 24 patients included, median age was 43.5 years (22–62) and a variety of donor types and stem cell sources were represented. At median follow-up of 26.9 months (2.4–72.7), no cases of grade III-IV acute (aGVHD) or severe chronic GVHD (cGVHD) were recorded. Viral reactivation was minimal, and there were no cases of graft failure or PTLD, with 100% disease-free and overall survival at last follow up. The estimate of 1-year GRFS was 86.3% (95% CI: 72.8–100%), with moderate cGVHD accounting for all events. The FCR regimen in SAA was well tolerated, even in older adults, with 100% disease-free survival with low GVHD and infection rates. These encouraging findings should be validated in larger prospective trials.

## Introduction

Acquired severe aplastic anemia (SAA) is a rare, life-threatening hematologic disorder resulting from an immune-mediated destruction of hematopoietic cells [1]. An inciting event provokes an aberrant immune response that leads to clonal expansion of cytotoxic T cells that target hematopoietic stem cells, resulting in hypocellular bone marrow and marked pancytopenia [2]. Existing treatment strategies aim to suppress or eradicate these pathogenic T-cell clones with either immunosuppressive therapy or via life-saving allogeneic hematopoietic cell transplantation (HCT) as a potentially curative therapy.

Newly diagnosed or relapsed/refractory SAA is an indication for allogeneic HCT regardless of age, and is favored as first-line for younger patients ( ≤ 40 years) who have an available matched related donor, while HCT is standardly withheld in older patients ( > 40 years) until failure of first-line immunosuppressive therapy [3, 4]. When HCT is used as a curative modality for SAA, bone marrow (BM) grafts are preferred, due to higher mortality and increased incidence of chronic graft-versus-host disease (cGVHD) with peripheral blood stem cell (PBSC) grafts [5]. Traditionally, an ablative conditioning regimen (typically consisting of cyclophosphamide 50 mg/kg for four doses in combination with anti-thymocyte globulin [ATG]) has been used routinely in younger patients, while the reduced intensity conditioning regimen (cyclophosphamide 30 mg/kg for four doses, fludarabine, ATG, and low-dose total body irradiation [TBI]) is commonly given for patients 40 years of age or older [6–9].

While varying conditioning strategies exist, there remains a distinct age effect in SAA following transplantation, with age remaining the most significant predictor of survival, particularly due to higher rates of graft failure and graft-versus-host disease (GVHD) in older patients [10]. Survival rates among patients who receive a matched related donor transplant for SAA are reported to be 82% in patients age 1–20 years, 72% in those 21–40 years, and 53% in those older than age 40 [1]. In addition to this age effect, known limitations in transplantation for SAA include the availability of preferred donor type and cell source (matched related donor and bone marrow, respectively), and relatively high rates of Epstein-Barr virus (EBV) reactivation and post-transplant lymphoproliferative disorder (PTLD), particularly with the use of ATG-containing conditioning regimens and/or cord blood grafts [11–13]. In an effort to decrease the risk of EBV-related PTLD, rituximab on day +5 following HCT for SAA has been introduced at a number of centers [1]. In a single-center study by Dominietto et al., a single dose of rituximab 200 mg at day +5 significantly lowered rates of EBV reactivation, as well as rates of grade II-IV GVHD compared to historical control among patients undergoing alternative donor HCT [14].

Based on this evidence, investigators at Vanderbilt University Medical Center (VUMC) and the associated Veterans Affairs hospital, Tennessee Valley Healthcare System (TVHS), previously published outcomes of a novel, non-myeloablative regimen consisting of fludarabine, cyclophosphamide, and rituximab (FCR) in 11 patients aged 40 years or older with SAA undergoing HCT [15, 16]. Results showed a 100% survival rate at a median follow-up of 302 days, as well as a 1-year GVHD-free/relapse-free survival (GRFS) rate of 81.8%, compared to 61.5% in a historical control group. These promising outcomes were associated with low toxicity rates, GVHD, and EBV reaction. Based on these positive results, this regimen was expanded to all patients, regardless of age, undergoing HCT for SAA at our institutions.

This study aims to evaluate the efficacy and safety of the FCR conditioning regimen among an expanded cohort of patients transplanted for SAA at VUMC and TVHS.

## Methods

### Study design

A dual-center, retrospective study of patients undergoing HCT for SAA was conducted at Vanderbilt University Medical Center (VUMC) and the associated Veterans Affairs hospital, Tennessee Valley Healthcare System (TVHS). The VUMC and TVHS Institutional Review Board approved the study. Patients with a SAA diagnosis who underwent first allogeneic HCT using FCR conditioning regimen at VUMC or TVHS between January 2016 and May 2022 were included in the study. Patients were excluded if they were younger than 18 or had not completed all planned treatments at the time of data collection.

### Preparative regimen for transplantation and GHVD

**Fig. 1:**
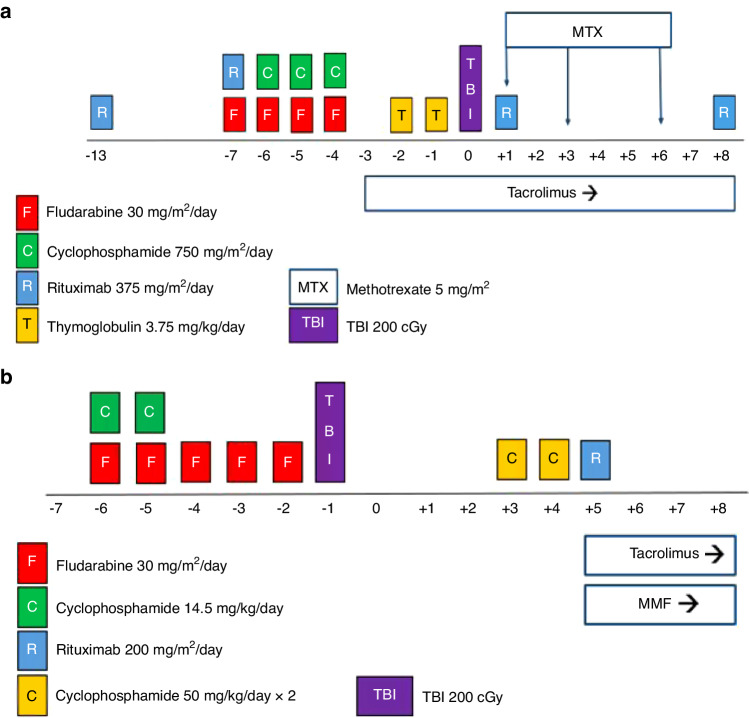
FCR conditioning regimen schemas. Doses and timing of each agent in FCR conditioning regimens for patients undergoing HCT for SAA with matched related, matched unrelated, or 1-allele mismatch donor (**a**) or haploidentical donor (**b**). Time in days is represented along the horizontal axis progressing from left to right, with day of transplantation depicted as day 0.

All patients received conditioning per established protocol as determined by degree of HLA-matching with their designated donor (Fig. 1). Patients received PBSC or BM grafts per treating physician’s discretion with allogeneic HCT performed on day 0. In patients with matched related, matched unrelated, or 1-allele mismatched donors, fludarabine (30 mg/m^2^) was given intravenously for four days (–7 to –4, i.e., 7 to 4 days before transplantation) in combination with cyclophosphamide (750 mg/m^2^) given intravenously for three days (–6 to –4) and anti-thymocyte globulin (rabbit) (3.75 mg/kg) for two days (–2 and –1). They were also given rituximab (375 mg/m^2^) on days –13, –7, +1, and +8.

Patients who underwent HCT from a haploidentical donor received fludarabine (30 mg/m^2^) intravenously for five days (–6 to –2) and cyclophosphamide (14.5 mg/kg) for two days (–6 to –5). They also received rituximab (200 mg/m^2^) on day +5. All patients received total body irradiation at a dose of 200 cGy on day 0 for those with matched related, unrelated, or 1-allele mismatched donors, or on day –1 for those with haploidentical donors.

Prophylaxis for GVHD in patients with matched related, matched unrelated, or 1-allele mismatch donors consisted of tacrolimus starting on day –3 and methotrexate, 5 mg/m^2^ intravenously, on days +1, +3, and +6 after transplantation. Patients with haploidentical donors received standard post-transplant cyclophosphamide (50 mg/kg on days +3 and +4), and tacrolimus and mycophenolate mofetil starting on day +5. Tacrolimus levels were adjusted to a goal range of 5–15 ng/mL per institutional standard, and administered for 180 days, at which point a taper was initiated provided absence of GVHD. Mycophenolate mofetil was administered until day +35 in patients with haploidentical donors.

### Primary and secondary outcomes

The primary outcome of interest was GVHD-free/relapse-free survival. A patient was considered to have an event if they experienced moderate or severe GVHD (including both acute GVHD [aGVHD] and cGVHD), relapse, or death. If a patient experienced multiple events, the earliest event date was used as the time to event (e.g., if a patient had a diagnosis of both aGVHD and cGVHD, the earliest date of diagnosis was used). If a patient did not experience an event until the end of follow-up time (i.e., the last date the patient was seen in the clinic or lost to follow-up [unable to contact, transitioned care to another city, etc.]), it was censored. Acute and chronic GVHD were graded according to Glucksburg and 2014 National Institutes of Health consensus criteria, respectively [17, 18]. Secondary outcomes included time to engraftment, incidence of graft failure, incidence of GVHD, rate of viral reactivation, post-HCT disease status, and number of inpatient hospital days.

### Statistical analysis

Descriptive statistics were used to summarize the patient characteristics. Medians and interquartile ranges (IQRs) were used for continuous variables, while frequencies and percentages were used for categorical variables. Differences in patient characteristics were tested for using Wilcoxon rank sum tests for continuous variables and chi-square tests for categorical variables. Probability of GRFS over time was estimated using the Kaplan-Meier estimation method.

## Results

### Study population

Between January 1, 2016 and May 31, 2022, 24 patients underwent HCT for SAA with the FCR conditioning regimen. Baseline characteristics for the cohort are described in Table 1. The median age among the study population was 43.5 years old (22–62), 42% of patients were female, and 83% were White. The median time from diagnosis to transplant was 14.5 months (2.8–213.6). The median Charlson comorbidity index was 0, and the median number of lines of therapy prior to transplant was 1.5, with all patients receiving at least one prior line of therapy. The most common treatments prior to HCT included the combination of cyclosporin A with ATG with or without eltrombopag and/or a corticosteroid. With regard to donor type, 58% of patients received their graft from a matched unrelated donor, 33% from a matched related donor, and 8% from a haploidentical donor. The stem cell source in most cases was PBSC, with 38% of patients receiving BM grafts.

**Table 1:** Baseline characteristics for the study population.

Baseline Characteristic	Patients *(N* = *24)*
Median duration of follow-up, days (range)	807 (72–2180)
Age at time of Transplant, median (IQR)	43.5 (33–52)
Female sex, no. (%)	10 (42)
White race, no. (%)	20 (83)
Median Charlson Comorbidity Index, score (range)	0 (0–5)
Median prior lines of therapy, no. (range)	1.5 (1–5)
Donor type, no. (%)	
• Haploidentical	2 (8)
• Matched related donor	8 (33)
• Matched unrelated donor	14 (58)
Stem cell source, no. (%)	
• Bone marrow	9 (38)
• Peripheral blood	15 (62)

### Primary and secondary outcomes

All outcomes are described in Table 2. 100% of patients were alive and in remission at a median time to last follow-up of 807 days (72–2180). The median time to engraftment for neutrophils and platelets was 14 days (10–25) and 18 days (10–38), respectively. There were no cases of graft failure recorded. The FCR regimen was administered in the outpatient HCT unit and the median number of inpatient hospital days through day +100 was 5 days (0–21).

**Table 2:** Description of secondary outcomes.

Outcome	Patients (*N* = *24*)
Survival, no. (%)	24 (100)
Median time to engraftment, days (range)	
• Neutrophils	14 (10–25)
• Platelets	18 (10–38)
Graft failure, no. (%)	0 (0)
Acute GVHD, no. (%)	
• Grade I	3 (13)
• Grade II	8 (33)
• Grade III-IV	0 (0)
Chronic GVHD, no. (%)	
• Mild	7 (29)
• Moderate	3 (13)
• Severe	0 (0)
Viral reactivation, no. (%)	
• CMV	1 (4)
• EBV	1 (4)

Acute GVHD was reported in 11 patients (grade I, *N* = 3, 13%; grade II, *N* = 8, 33%). No patients experienced grade III-IV aGVHD. The most common site of aGVHD was the skin (*N* = 7) and gastrointestinal (GI) tract (*N* = 6), and only 1 patient experienced aGVHD of the liver. The median time to onset of aGVHD was 48 days post-HCT. Chronic GVHD was reported in 10 patients, with a median time to onset of cGVHD of 184 days. Seven patients experienced mild cGVHD, while 3 patients experienced moderate cGVHD. There were no reported cases of severe cGVHD. The most common sites of cGVHD were oral and skin, both occurring in 4 patients, followed by ocular in 3 patients, GI in 2 patients, and possible liver reported in 1 patient. Of the patients who experienced aGVHD, 7 received PBSC, while 4 received BM grafts, and of the patients who experienced cGVHD, 5 received PBSC, and 5 received BM grafts. There were no cases of steroid-refractory acute or chronic GVHD. There were no significant differences in cell source or baseline characteristics between patients who experienced GVHD and those who did not.

By time of last follow-up, only 2 patients experienced viral reactivation at any point. One patient experienced reactivation of CMV on day +35 following the transplant, which was treated with ganciclovir. One patient experienced laboratory EBV reactivation over 400 days after the transplant, which resolved without intervention.

**Fig. 2:**
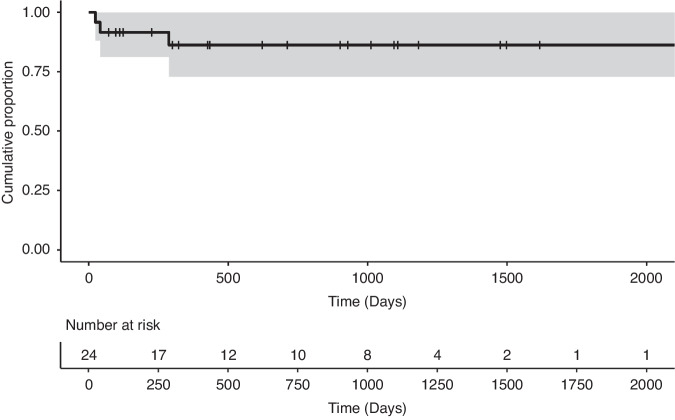
GVHD-free/relapse-free survival. The Kaplan–Meier curve for GVHD-free/relapse-free survival. The Kaplan–Meier estimate of 1-year GRFS was 86.3% (95% confidence interval: 72.8–100%). Moderate cGVHD accounted for all GRFS events.

The Kaplan-Meier estimate of 1-year GRFS was 86.3% (95% confidence interval: 72.8–100%) (Fig. 2), with moderate cGVHD accounting for all GRFS events in the study cohort.

## Discussion

This study found that a non-ablative FCR conditioning regimen for allogeneic HCT for SAA was associated with no transplant-related mortality and 100% disease-free survival, and very low rates of GVHD, including among older adults.

Prior to implementation of the FCR regimen, our historical SAA population exhibited an estimated 1-year GRFS of 61.5% [16]. The first 11 patients to receive the FCR regimen at VUMC demonstrated an estimated 1-year GRFS of 81.8% [16]. This study represents over twice as many patients as the previous review and demonstrates a sustained to slightly improved 1-year GRFS of 86.3%, with longer overall follow-up and sustained 100% disease-free survival. The population enrolled in this expanded cohort represents a high-risk population undergoing HCT for SAA, with a median age greater than 40 years old, majority having matched unrelated donors and peripheral blood stem cell source. Moreover, previous data demonstrate that an interval of two years or less between diagnosis and HCT is a significant factor impacting survival, and 33% of patients in this cohort underwent HCT more than two years after diagnosis of SAA [19]. Despite this high-risk population, rates of complications, including cGVHD and infections, remained low, and in cases where they did occur, overall morbidity was non-severe. Furthermore, this study reinforces the ability to administer the conditioning regimen in the outpatient setting, with all non-haploidentical transplants at VUMC administered entirely in the outpatient setting and minimal need for hospitalization within 100 days of transplantation.

In this study, 46% of patients experienced aGVHD and 42% of patients experienced cGVHD. However, all patients who experienced aGVHD developed only low-grade (I-II) aGVHD; of the patients who experienced cGVHD, the majority reported only mild symptoms. When comparing GRFS events to historical cohorts, death without relapse or GVHD accounted for the majority of events in the historical cohort, while grade III acute GVHD in 2 patients accounted for all events in the first 11 patients reported on, and moderate cGVHD accounted for the 3 GRFS events in this cohort. The repeated demonstration of low rates of GVHD contributing to all GRFS events in this population continues to support the efficacy and tolerability of the FCR regimen.

EBV reactivation and PTLD are well-known post-HCT complications that have been seen at higher rates in patients with SAA compared to the broader HCT population despite many centers incorporating a single dose of rituximab into their standard regimens in recent years [1, 13]. This study did not report such complications, with no patients experiencing PTLD, and only 1 patient experiencing minor elevation of EBV viral load, which did not require intervention. Based on these findings, the incorporation of three additional doses of rituximab at approximately weekly intervals for patients with matched related, matched unrelated, or 1-allele mismatch donors continue to demonstrate benefit in decreasing the likelihood of EBV reactivation and may also contribute to enhanced GVHD prophylaxis and prevention of disease relapse via immunomodulation.

This study is not without limitations, including the retrospective nature of the analysis conducted at only two centers. Despite representing the largest cohort of patients to receive HCT for SAA utilizing the FCR regimen, this remains an overall small sample size. The population is also quite heterogeneous, and the length of follow-up time had a wide range and much variation among patients. Despite these limitations, this study offers continued positive findings in a high-risk cohort. The possibility of limited complications and no transplant-related mortality while utilizing alternative donors and PBSC as stem cell source is groundbreaking in a population in which these options had previously been thought to play a limited role. While this cohort represented a variety of donor types and stem cell sources, only 2 patients underwent transplantation with a graft from a haploidentical donor. Though no efficacy or safety outcomes were observed at an increased rate in any specific donor type, this small population of haploidentical transplants is a significant limitation as the role of alternative donors is an area of great interest for the SAA population. The expansion of donor pools and stem cell sources may allow for an increased number of transplants overall in the SAA population and shorten the time to transplantation, which may also have implications for overall survival given the high risk of infection and mortality in the SAA population.

## Conclusion

This study demonstrated 100% disease-free survival and low rates of complications utilizing a novel, non-ablative FCR conditioning regimen for HCT in SAA, even in older adults and patients with significant co-morbidities. Despite our study limitations, further prospective validation in a larger SAA population is warranted to fully investigate this regimen’s potential role as standard of care.

## Data Availability

Dataset is available from the corresponding author on reasonable request.
